# Improving quality of medical certification of causes of death in health facilities in Tanzania 2014–2019

**DOI:** 10.1186/s12913-021-06189-7

**Published:** 2021-09-13

**Authors:** Trust Nyondo, Gisbert Msigwa, Daniel Cobos, Gregory Kabadi, Tumaniel Macha, Emilian Karugendo, Joyce Mugasa, Geofrey Semu, Francis Levira, Carmen Sant Fruchtman, James Mwanza, Isaac Lyatuu, Martin Bratschi, Claud J. Kumalija, Philip Setel, Don de Savigny

**Affiliations:** 1 Ministry of Health, Community Development, Gender, Elderly, and Children, Dodoma, Tanzania; 2 Bloomberg Philanthropies Data for Health Initiative, Vital Strategies, New York, NY USA; 3 Swiss Tropical and Public Health Institute, University of Basel, Basel, Switzerland; 4 National Bureau of Statistics, Dodoma, Tanzania; 5 Muhimbili National Hospital, Dar es Salaam, Tanzania; 6 Ifakara Health Institute, Dar es Salaam, Tanzania; 7 Africa Academy for Public Health, Dar es Salaam, Tanzania

**Keywords:** Data quality assessment, Mortality data, Causes of death, ICD-10, Start-up mortality list, DHIS2, Continuing professional development, Medical certification, eHealth, Civil Registration and vital statistics, Tanzania

## Abstract

**Background:**

Monitoring medically certified causes of death is essential to shape national health policies, track progress to Sustainable Development Goals, and gauge responses to epidemic and pandemic disease. The combination of electronic health information systems with new methods for data quality monitoring can facilitate quality assessments and help target quality improvement. Since 2015, Tanzania has been upgrading its Civil Registration and Vital Statistics system including efforts to improve the availability and quality of mortality data.

**Methods:**

We used a computer application (ANACONDA v4.01) to assess the quality of medical certification of cause of death (MCCD) and ICD-10 coding for the underlying cause of death for 155,461 deaths from health facilities from 2014 to 2018. From 2018 to 2019, we continued quality analysis for 2690 deaths in one large administrative region 9 months before, and 9 months following MCCD quality improvement interventions. Interventions addressed governance, training, process, and practice. We assessed changes in the levels, distributions, and nature of unusable and insufficiently specified codes, and how these influenced estimates of the leading causes of death.

**Results:**

9.7% of expected annual deaths in Tanzania obtained a medically certified cause of death. Of these, 52% of MCCD ICD-10 codes were usable for health policy and planning, with no significant improvement over 5 years. Of certified deaths, 25% had unusable codes, 17% had insufficiently specified codes, and 6% were undetermined causes. Comparing the before and after intervention periods in one Region, codes usable for public health policy purposes improved from 48 to 65% within 1 year and the resulting distortions in the top twenty cause-specific mortality fractions due to unusable causes reduced from 27.4 to 13.5%.

**Conclusion:**

Data from less than 5% of annual deaths in Tanzania are usable for informing policy. For deaths with medical certification, errors were prevalent in almost half. This constrains capacity to monitor the 15 SDG indicators that require cause-specific mortality. Sustainable quality assurance mechanisms and interventions can result in rapid improvements in the quality of medically certified causes of death. ANACONDA provides an effective means for evaluation of such changes and helps target interventions to remaining weaknesses.

**Supplementary Information:**

The online version contains supplementary material available at 10.1186/s12913-021-06189-7.

## Background

There have been significant gains in population health and survival in Africa including Tanzania since 2000 [1–3]. In two decades, under-five mortality in Tanzania declined from 141 to 67 per 1000 live births, crude death rate declined from 14 to 6.5 deaths per 1000 and life expectancy at birth increased from 53 to 68 years) [4]. These changes coincide with changes in both the cause mix and cause-specific fractions of the major causes of death. We know less about the inequalities and gender dimensions of these dynamics [5, 6].

Monitoring mortality and cause of death is essential to shape national health policies, to track progress to the 2030 Sustainable Development Goals (SDGs), and to gauge responses to pandemic disease. Understanding how mortality patterns and their underlying risk factors are changing requires timely empirical data of sufficient scale (representativeness), quantity, and quality to inform national health policy [7, 8]. Moreover, Tanzania plans to report its annual progress toward Sustainable Development Goals (SDGs) for the 15 SDG indicators that require cause-specific mortality data. The preferred source for empirically derived causes of death is medical certificates of cause of death for deaths occurring in health facilities, mainly hospitals, and from the medico-legal death investigation system, where physicians ascertain the cause of death (COD) [9]. Civil Registration and Vital Statistics (CRVS) systems are key instruments for such data [10].

Tanzania has been upgrading and decentralizing its CRVS system since 2015 [11]. Part of this effort focuses on improving the availability and quality of cause of death data produced by health facilities reporting to the Ministry of Health, Community Development, Gender, Elderly and Children (MoHCDGEC). Since 2003, Tanzania has required a WHO standardized Medical Certificate of Cause of Death (MCCD) certified by a clinician for all clinician-attended deaths in a public or private health facility [12]. Tanzania’s population of over 55 million in 2019 experienced an estimated 362,000 annual deaths according to the National Bureau of Statistics [4]. Health facility-based medical doctors are currently the primary source of disease-specific mortality evidence needed for monitoring population health and national health policies. Globally, Tanzania has one of the lowest densities of medical doctors (0.4 per 10,000 population) [13].

**Fig. 1:**
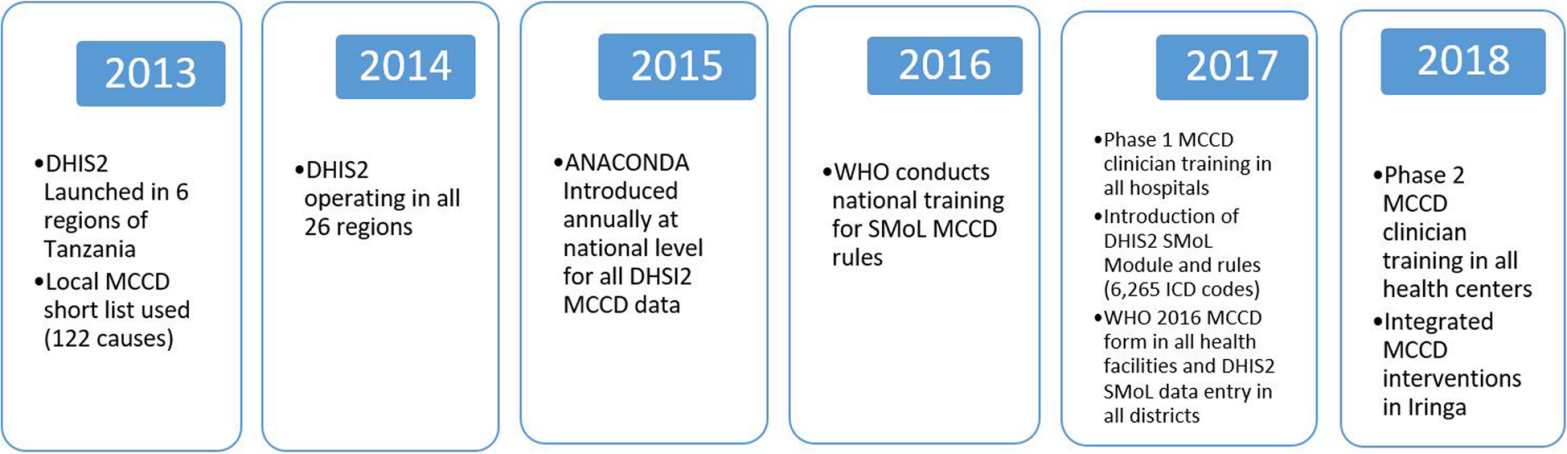
Timeline of relevant events

A digital database of hospital deaths recording location, year, age, sex, and ICD-10 code for the underlying medically certified cause of death has been available in the MoHCDGEC nation-wide since 2014 in its District Health Information System (DHIS2) (Fig. 1).

Training on medical certification of cause of death among health care providers elsewhere has shown positive effects on improving the quality of MCCDs and, consequently, on the quality of country mortality statistics [14–16]. In late 2018, Tanzania launched a package of linked interventions aimed specifically at improving the quality of medical certification in all health facilities in Iringa Region. Iringa is one of 26 administrative regions of Tanzania with a population of 1,066,695 in 2018 and an estimated 6800 deaths per year [4]. Tanzania used a Theory of Change to inform the mix of interventions beyond training applied in Iringa Region to improve medical certification in health facilities.

The Iringa interventions provided an opportunity to assess the combined effect of such interventions. Therefore, we applied the most recently available quality assessment tool for medically certified causes of death to the last 5 years of the national cause-specific mortality dataset for baseline context, and the pre- and post-intervention dataset in Iringa Region for intervention effect.

## Methods

### Setting and study population

**Fig. 2:**
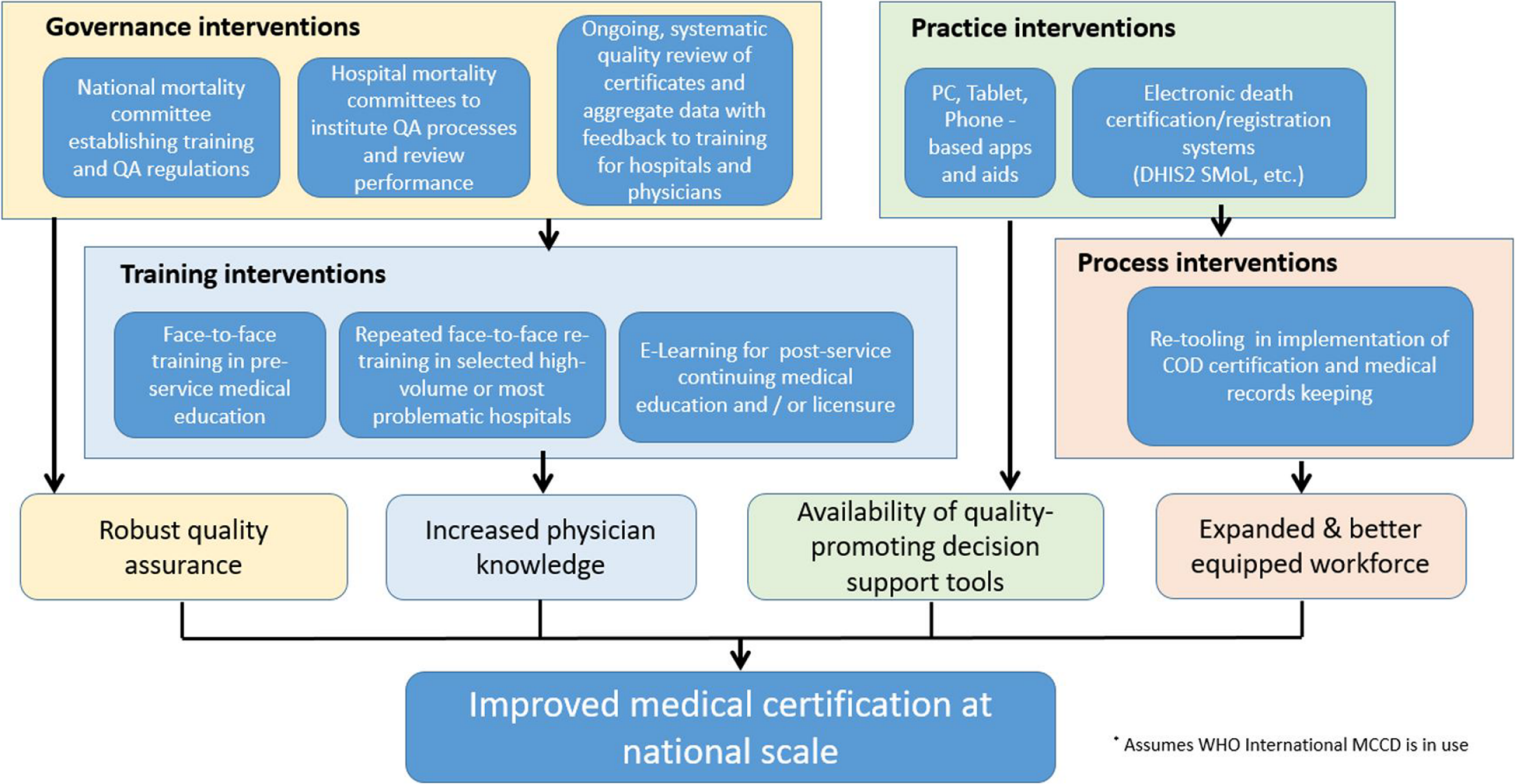
Theory of change for improving MCCD quality

This report is an observational study that assesses the baseline quality of medical certification of cause of death data at the national level from 2014 to 2018 during a period of no specific interventions on MCCD quality and further documents the combined effect of a suite of interventions drawn from a theory of change (Fig. 2) on the quality of MCCD in one Region between 2018 and 2019 in a non-controlled before and after study. The study populations are all deaths in health facilities that received a medical certificate of cause of death entered in DHIS2.

Tanzania is a low-income country with an annual GDP and total health expenditure per capita of $2861 and 104 USD (PPP) respectively in 2017 [17]. The Tanzania mainland has a council (district and municipal administrative areas) decentralized health system extending to 184 councils. There are a total of 8670 registered health facilities. At tertiary and secondary levels, there are 294 hospitals (including teaching, referral and specialist hospitals), 28 regional hospitals, 139 district hospitals, 57 private hospitals, and 910 health centers, all with in-patient facilities and physicians on staff who are obligated to certify deaths. At primary care levels, there are 7242 government, NGO, and private dispensaries usually managed by clinical officers who may also certify deaths. This study draws on health facility data from 8107 DHIS2 reporting health facilities nation-wide (94% of all health facilities) from 2014 to 2018 including Iringa Region in Central Tanzania receiving specific MCCD improvement interventions from 2018 to 2019.

### Health facility cause of death data

Since 2014, trained clinicians and health information staff certify and code deaths according to the rules of the WHO International Statistical Classification of Diseases and Related Health Problems, 10th Revision (ICD-10) [18]. The WHO-based MCCD (paper form) in use in Tanzania documents the sequence, timing, and context of morbid events leading to death and is required to be completed by physicians. This form records the immediate, intermediate and underlying causes of each death (COD) and contextual circumstances. The underlying cause of death (uCOD) is the cause in the sequence of causes that initiates the chain of events leading to death.

As part of routine HMIS, district-level health information officers check and compile these causes with associated codes from the MCCD paper forms from hospitals and lower-level health facilities and enter them as individual records into an electronic District Health Information System (DHIS2). DHIS2 is an integrated decentralized electronic Health Management Information system that includes a function to record and report individual causes of death in health facilities. These data are entered at the district level using an online browser that is linked to the central server of the MoHCDGEC at the national level. From the central server, the data are continuously available on a restricted basis to designated authorized staff of the MoHCDGEC and lower levels of the health system.

Until 2018, Tanzania used its own customized ICD-10 shortlist dictionary of 122 possible causes of death with up to 487 potential ICD-10 coded causes in sub-categories [19] in its DHIS2. In 2019, there was a phased introduction Nation-wide and in Iringa Region of the WHO ICD-10 SMoL DHIS2 module with a dictionary of 1254 potential ICD-10 codes [20]. For this study, DHIS2 provided annual export files containing line listings of the uCOD text, ICD-10 (3 and 4 digits) code, sex, and exact age at death for each death.

### Completeness of cause of death recording based on medical certificates of deaths

The proportion of observed versus expected deaths (sometimes referred to as completeness regarding registered deaths) is a key indicator of data quality. We calculated completeness for the medically certified death for each year at national level based on a simple aggregated analysis comparing the observed deaths with the expected deaths estimated as the product of the annual population estimates times the annual crude death rates from the National Bureau of Statistics [21, 22]. We also used ANACONDA to calculate a modelled estimate of the proportion of observed versus expected deaths for males and females separately. The latter was done using the Adair-Lopez Method [23]. This is an indirect demographic method based on a random effects statistical model that estimates sex-specific completeness as a function of its relationship with empirical inputs for the observed crude death rate, the observed under-5 mortality rate, and the observed proportion of individuals aged 65 years and older, plus a reliable estimate of the true under-5 mortality rate (UN Inter-agency Group for Child Mortality Estimation). We provide both the simple and the modelled estimates of the proportion of expected deaths at the national level having an MCCD.

### Mortality data quality assessment

We used a computer application (ANACONDA version 4.0.1) [24] to assess the quality of data on age, sex and ICD-10 code for the underlying cause of death. There were 155,461 medically certified deaths at the national mainland level between 2014 and 2018 and in Iringa Region there were 1543 medically certified deaths over 9 months before the interventions (October 2017 to June 2018) and 1147 deaths over 9 months post-intervention (October 2018 to June 2019). There was an intervening four-month burn-in period for interventions.

The Tanzania National Bureau of Statistics provided population denominators by sex and age for 0-11 months, 1–4 years and 5-year age groups from 5 years through to age group 80+ [4]. We entered denominator population data and corresponding numerator uCOD ICD-10 codes for deaths in these sex and age groups into the ANACONDA data template. We uploaded the template data into ANACONDA to create the annual workspace files for analyses (available as supplemental files). ANACONDA then performed 28 automated data-quality assessments [25] and provided tabulations and graphics on age and sex-specific mortality rates and causes of death.

### Classification of underlying cause of death data errors

ANACONDA identifies causes that are invalid as underlying causes of death according to ICD rules. These are usually termed “garbage” ICD-10 codes [26–28]. In this paper, we call them unusable codes. ANACONDA groups these unusable codes into four categories according to ICD concepts (Table 1). ANACONDA also assesses a fifth category that includes valid codes for causes that are insufficiently specified for optimal use as uCOD.

**Table 1:** Categories of unusable and insufficiently specified ICD-10 codes

**Category 1: “Symptoms, signs and ill-defined conditions as the cause of death”** mostly drawn from the ICD-10 Chapter XVIII codes or R codes.	
**Category 2: “Impossible as underlying causes of death”** including conditions such as essential hypertension and atherosclerosis as well as causes which are the long-term sequelae of various diseases.	
**Category 3: “Intermediate causes of death”** being diseases or injuries that have been precipitated by an underlying cause (e.g. heart failure).	
**Category 4: “Immediate causes of death”** such as cardiac arrest or respiratory failure.	
**Category 5: “Insufficiently specified causes of death within ICD Chapters”** partially informative causes such as causes within a larger cause of death category, e.g. malignant neoplasm without specification of site, or unspecified vehicle accidents.	

ANACONDA also categorizes the unusable and insufficiently specified ICD-codes into four levels of severity according to their potential impact in misdirecting policy and planning [28]. We show how causes with Severity Levels 1, 2 and 3 (very high, high, and medium severity) distort the tabulation of the top 20 causes of death.

### MCCD interventions in Iringa region

The MoHCDGEC applied a suite of interventions drawn from a theory of change aimed at improving medical certification of cause of death. The theory of change (Fig. 2) indicates how these approaches could work synergistically to improve MCCD quality. The interventions from the theory of change that were applied in Iringa include:

1. **Governance interventions.** These included the establishment of a National Mortality Committee, hospital mortality committees, and quality reviews of nationally assembled data using ANACONDA. A National Mortality Surveillance training team started in 2016 and included experts from the MoHCDGEC, the Muhimbili National Hospital and the Northern Zonal Referral Hospital (Kilimanjaro Christian Medical Centre). At the regional level, Iringa benefited from these, plus the inputs from the quality reviews using ANACONDA into their training interventions.
2. **Training interventions.** At national level, in August 2018, the MoHCDGEC initiated comprehensive nationwide training on MCCD to help clinicians complete MCCD forms more correctly [19]. Training included pre- and post-service face-to-face MCCD training supplemented by access to an e-Learning tool for post-service continuing professional development (CPD) [29]. Training was by an interactive adult learning model with exercises to equip all participants with knowledge for selecting the proper underlying causes of death and assignment of ICD-10 codes. In Iringa Region, the training was modified such that it covered all clinicians (524 clinicians, 334 male and 190 female) (see task delegation below in Process Interventions), unlike other regions where only two clinicians were trained in each tertiary or secondary health facility (i.e. hospitals and health centers).
3. **Practice interventions.** In Iringa Region, these included electronic data entry of the WHO MCCD form in DHIS2. In January 2019, the Ministry introduced a WHO software application as part of its DHIS2 that uses the WHO Start-up Mortality List (SMoL) giving on-line access to a dictionary of 6265 possible morbid conditions (including aliases). The MCCD SMoL module in DHIS2 is such that the electronic data entry form mimics the MCCD paper form and the cause list is in the format of a drop-down pick-list where data entry staff can select the underlying, intermediate and immediate causes of death. Furthermore, as part of the DHIS2 SMoL Training Program, 26 simplified rules for selecting the uCOD are applied by the clinician and the data entry staff to identify the uCOD [30]. The DHIS SMoL module automatically assigns one of 1254 distinct ICD-10 codes to each of the available possible morbid events reported on the MCCD form as data entry occurs in DHIS2.
4. **Process interventions.** In Iringa Region, these included MCCD task delegation to all clinician cadres (excluding nurses) and revised standard operating procedures and processes for completing MCCD forms and record processing. The task sharing targeted by MCCD training delegated across Medical Doctors, Assistant Medical Officers, Clinical Officers, Assistant Clinical Officers and Medical Specialists (excluding nurses) working in all tertiary, secondary and primary health facilities, both government and non-governmental. The training was accordingly extended to Regional Medical Officers, District Medical Officers, Regional Health Management Information System Focal Persons, District Health Management Information System Focal Persons, and Health Facility Medical Officers in Charge, who were commissioned to oversee and ensure data quality under their respective authorities.

Interventions described above that were not implemented at national level between 2014 and 2018 include the more intensive training of all clinicians rather than only two per facility, the task delegation for medical certification, and the use of DHIS2 with the SMoL mortality module.

## Results

### Completeness of cause of death recording based on medical certificates of death

The proportion of deaths with an MCCD at national level, calculated using the annual crude death rate per annual national mainland population shows an average of 9.7% of the estimated number of annual deaths in Tanzania received an ICD-10 code of any quality (range 8.5 to 10.4%). The value calculated from the Adair-Lopez ANACONDA data model [23] similarly shows an estimated average proportion over the same years for males and females separately (9.1% for males and 12.2% for females). There was no clear trend or improvement in the proportion of deaths certified over the 5 years from 2014 to 2018 (see supplemental material). Thus, cause of death data of any quality is currently only available for 9 to 12% of the estimated number of expected annual deaths in Tanzania up to 2018.

### ICD-10 code ranges

Our ANACONDA national analyses for the years 2014–2018 inclusive showed an average of 151 distinct ICD-10 codes used per year (range 88 to 305). However, the number of discrete ICD-10 codes seen in the pre-and post-intervention datasets in Iringa increased substantially from 110 codes to 482 codes, suggesting a potential for a greater degree of specificity in coding post-intervention (Table 2).

**Table 2:** Distribution of causes of death by broad categories and usability at national level in each year and in one region before and after MCCD quality improvement interventions

	National level						Iringa intervention region	
Year	2014	2015	2016	2017	2018	Mean	2018 Pre-	2019 - Post
Number of deaths	22,935	35,508	34,189	36,631	26,198	31,092	1543	1147
Unique ICD Codes	99	108	109	119	324	152	110	482
Group 1 Communicable	49.0%	43.8%	42.9%	41.6%	40.8%	43.6%	38.9%	46.6%
Group 2 NCDs	8.6%	6.8%	6.9%	7.0%	8.6%	7.6%	7.5%	15.3%
Group 3 External	0.0%	0.6%	0.9%	0.8%	1.0%	0.7%	1.9%	3.0%
Unknown	0.8%	11.1%	6.4%	9.4%	5.4%	6.6%	3.6%	3.0%
Unusable Codes	20.4%	22.0%	24.7%	26.8%	29.3%	24.6%	28.0%	23.2%
Insufficiently Specified causes	21.2%	15.7%	18.0%	14.4%	14.9%	16.8%	20.2%	9.0%
	100%	100%	100%	100%	100%	100%	100%	100%
Overall % Usable Causes	57.6%	51.2%	50.8%	49.4%	50.4%	51.9%	48.2%	64.9%

Unusable codes are combined from Category 1 to 4; Insufficiently specified causes are from Category 5

### Distribution of causes of death by broad categories and by usability category

In Table 2, we provide the total numbers of deaths and the proportions according to the Global Burden of Disease (GBD) broad cause groups. Over the 5 years the proportion in Group 1, communicable, maternal, neonatal and nutritional causes declined from 49 to 41%. Group 2, non-communicable diseases showed little change averaging at 7.6% and Group 3, external causes due to injury remained unexpectedly low and consistently 1% or less. Unusable codes (Categories 1 to 4) accounted for some of the variability with a mean of 24.6% (range 20.4 to 29.3%) while insufficiently specified codes (Category 5) accounted for a mean of 16.8% of deaths (range 14.4 to 21.2%).

We also examined the biologically implausible causes of death each year. These are deaths with valid usable ICD-10 codes but with an implausible age and or sex. Examples include maternal hypertensive disorders in males or neonatal encephalopathy in ages over 10 years. These rates were consistently low and relatively unchanging over the five-years 2014–2018 at 4.4, 3.6, 4.6, 4.3 and 3.1% with an average of 4.0%.

Of all deaths, the mean proportion that had underlying causes that were fully usable for public health purposes was 51.9%, (range 49.4 to 57.6%). The remainder (48.1%) were unknown, unusable, or insufficiently specified. Over the 5 years, there is no significant trend in quality.

### Types of unusable causes

An analysis of the 65,363 deaths with unusable codes in categories 1 to 5 across the 5 years (Table 3) shows that physicians provided sufficient information and ICD mortality coders were correct in never selecting the immediate cause of death as the uCOD (Category 4). This is consistently so in every year. Similarly, there was little problem with Category 1 (Symptoms, signs and ill-defined COD) with a mean of 2.5% of the unusable codes (range 0.2 to 11.4%), and with Category 2 (Impossible underlying COD) with a mean 1.7% of unusable codes (range 0.9 to 3.4%). The majority of the unusable codes were either Category 3 (Intermediate COD) (mean of 37.0%, range 29.8 to 43.3%) or Category 5 (Insufficiently specified COD) (mean 58.9%, range 50.2 to 66.5%). However, there is an unexplained increase in Category 1 causes in 2018 which needs to be followed up in subsequent years.

**Table 3:** Number of deaths at national level with unusable codes and percent in each category of unusable code

Year	Number of deaths with unusable code	Signs, symptoms, Ill-defined COD (Cat 1)	Impossible COD (Cat 2)	Intermediate COD (Cat 3)	Immediate COD (Cat 4)	Insufficiently specified COD (Cat 5)	% Total unuseable codes
2014	9536	0.3%	3.4%	29.8%	0.0%	66.5%	100%
2015	14,531	0.3%	1.4%	37.3%	0.0%	61.1%	100%
2016	14,607	0.2%	0.9%	37.6%	0.0%	61.3%	100%
2017	15,115	0.4%	1.0%	43.3%	0.0%	55.4%	100%
2018	11,574	11.4%	1.6%	36.8%	0.0%	50.2%	100%
Mean	13,073	2.5%	1.7%	37.0%	0.0%	58.9%	100%

### Specific unusable ICD-10 codes with high frequency at national level in 2018

In Table 4, we extract the 15 most frequent unusable ICD-10 codes and causes in 2018 in Category 1–4 inclusive. These are for 6008 deaths, which is 78% of the total 7676 deaths with Category 1–4 unusable codes. The frequency distribution skews such that the three highest-ranked unusable causes comprise almost one-half of these unusable codes. Specifically, these are ICD code I10 - essential primary hypertension; R99 - other ill-defined and unspecified causes of mortality; and I50 - heart failure. The five most common unusable codes are also seen at high frequency in the prior years’ data sets.

**Table 4:** Frequency of the 15 most common unusable ICD-10 codes in national DHIS2 health facility data for 2018

ICD-10 code	Cause	Number of deaths
I10.	Essential (primary) hypertension	1281
R99.	Other ill-defined and unspecified causes of mortality	1261
I50.	Heart failure	1131
A41.	Other septicemia	884
S09.	Other and unspecified injury of the head	290
D64.	Other anemias	213
C80.	Malignant neoplasm without specification of site	207
J06.	Acute upper respiratory infection of multiple unspecified sites	192
J81.	Pulmonary oedema	160
D50.	Iron deficiency anemia	112
K65.	Peritonitis	92
R57	Shock	73
R50.	Fever of unknown origin	51
N17.	Acute renal failure	37
X49.	Accidental poisoning and or exposure to other and unspecified chemicals and toxins	24
	Total	6008

### Distribution of causes before and after intervention in Iringa region

**Fig. 3:**
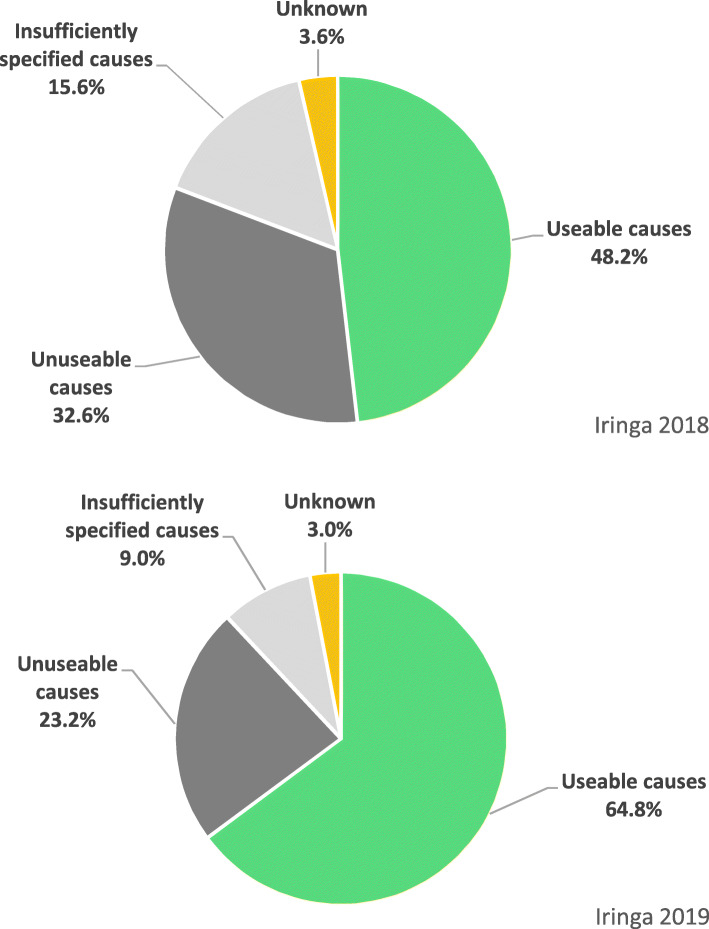
Distribution of usable, unusable (garbage), insufficiently specified, and unknown causes before (top) and after (bottom) the MCCD interventions in Iringa Region

There was an important reduction in the proportion of ICD-10 codes deemed as unusable codes from 32.6 to 23.2% pre- and post-intervention respectively (Fig. 3). The proportion of insufficiently specified codes also reduced from 15.6 to 9.0%. The resulting redistribution of the additional usable codes to the three Global Burden of Disease (GBD) broad causes meant that the broad cause shares changed concordantly with increases across all three broad causes. However, Group II (NCDs) increased the most. Group III (Injury) increased from 1.9 to 3.0% but remains unusually low. Biologically implausible causes which are causes that have valid usable ICD-10 codes but with implausible age and/or sex were reduced from 2.4 to 1.3% of medically certified deaths. The net effect is that policy usable causes increased significantly from 48.2% (CI 45.7–50.7%) to 64.9% (CI 62.1–67.6%) (Fig. 3).

### Distribution of unusable causes before and after intervention in Iringa region

There was a reduction in the frequency of unusable causes across all five categories of usability. The greatest proportionate reductions were in the frequency of Category 1 - Unusable symptoms, signs, and ill-defined conditions (7.2 to 3.7%), Category 3 - Unusable intermediate causes (18.1 to 12.2%) and in Category 5 - Usable but insufficiently specified causes (20.2 to 13.9%).

### Implications of unusable causes in the top 20 causes in Iringa region

The frequency of unusable and insufficiently specified codes influences the top twenty cause lists. Such unusable causes decreased from 11 to 7 out of 20 before and after training respectively in both males and females (females shown in Table 5). Most of the unusable code item reduction was due to a reduction in unusable causes with low severity, however, the ranks of unusable codes shifted such that there was an important decline in the cumulative cause-specific mortality fractions due to high impact unusable codes from 27.4 to 13.5% before and after interventions respectively.

**Table 5:** Ranking of 20 leading causes of deaths for females in health facilities before (top) and after (bottom) MCCD interventions in the Iringa Region

Rank	CSMF (%)	ICD code	Severity	Cause
1	11.9	B24		Unspecified human immunodeficiency virus [HIV] disease
2	7.8	R99	H	Other ill-defined and unspecified causes of mortality
3	6.8	J18	L	Pneumonia, organism unspecified
4	6.5	I10	H	Essential (primary) hypertension
5	3.9	I42	L	Cardiomyopathy
6	3.5	A41	H	Other septicaemia
7	3.3	P95		Fetal death of unspecified cause
8	3.0	I50	H	Heart failure
9	2.9	A09		Diarrhoea and gastroenteritis of presumed infectious origin
10	2.9	Z21	H	Asymptomatic human immunodeficiency virus [HIV] infection status
11	2.3	B45		Cryptococcosis
12	2.3	P21		Birth asphyxia
13	2.2	P15		Other birth injuries
14	2.0	T71	H	Asphyxiation
15	1.8	P05		Slow fetal growth and fetal malnutrition
16	1.7	A15		Respiratory tuberculosis, bacteriologically & histologically confirmed
17	1.7	R50	H	Fever of unknown origin
18	1.6	E14	L	Unspecified diabetes mellitus
19	1.6	I64	L	Stroke, not specified as haemorrhage or infarction
20	1.3	B53		Other parasitologically confirmed malaria
Rank	CSMF (%)	ICD code	Severity	Cause
1	11.4	B22		Human immunodeficiency virus disease resulting in other specified diseases
2	6.0	J18	L	Pneumonia, organism unspecified
3	4.5	I50	H	Heart failure
4	4.0	B24		Unspecified human immunodeficiency virus [HIV] disease
5	4.0	P21		Birth asphyxia
6	3.4	I11		Hypertensive heart disease
7	3.1	B20		Human immunodeficiency virus [HIV] disease resulting in infectious diseases
8	2.7	B45		Cryptococcosis
9	2.7	D64	H	Other anaemias
10	2.3	R99	H	Other ill-defined and unspecified causes of mortality
11	2.2	I10	H	Essential (primary) hypertension
12	2.0	P95		Fetal death of unspecified cause
13	1.8	A41	H	Other septicaemia
14	1.8	P02		Fetus and newborn affected by complications of placenta, cord, membranes
15	1.6	P36		Bacterial sepsis of newborn
16	1.4	B59		Pneumocystosis
17	1.4	E46		Unspecified protein-energy malnutrition
18	1.4	P22		Respiratory distress of newborn
19	1.4	R57	H	Shock, not elsewhere classified
20	1.3	A18		Tuberculosis of other organs

H = Unusable codes with very high, high, or medium impact

L = Usable but insufficiently specified codes with low policy impact

## Discussion

There is growing interest in cause-specific mortality globally and in Tanzania given the increasing dynamics in population health threats, health equity, and the importance of reporting on SDG goals [31–35]. Geopolitical, ideological, technological, environmental and epidemiological conditions are changing rapidly. More than ever, countries need to monitor the quality of local mortality data and use their analyses to improve the coverage and quality of the data they generate.

For health facility data, there is a need to establish sustainable, routine, quality assurance and improvement systems. Data quality checks on mortality data at source with appropriate feedback loops to training would ideally exist at the facility level [36]. At national and sub-national (e.g. district/regional) routine audits with tools such as ANACOD or ANACONDA [16, 25] now exist to assess the quality of data from across the system.

### Availability of cause of death data

Our ANACONDA analysis of 155,461 health facility deaths over 5 years in Tanzania shows no trend between 2014 and 2018 in the proportion of annual deaths having an MCCD with uCOD (completeness) (see supplemental files). This period covers the first 5 years of the use of DHIS2. It suggests that merely having access to digital Health Management Information System data compared with paper-based data does not necessarily result in improved availability of appropriate COD data. We note that there was no specific intervention to improve data coverage or quality until 2018 so it is too early to detect intervention related improvement at national level. The national results reported here constitute a baseline and a context for the Iringa Region intervention effects.

The national analysis shows that approximately 10% of expected deaths in Tanzania occur in health facilities participating in DHIS2 and receive an MCCD. There was close correspondence in this measure both by direct estimation, and by the Adair-Lopez method [23]. This result is similar to current estimates from the MoHCDGEC [31] which concluded data is available on only 10% of deaths. Mremi et al. [37] recently explored the root causes of mortality data availability in Tanzanian hospitals for the period between 2006 and 2015 and found that MCCD ICD-10 forms were available in hospitals only after 2013 and used in only 28% (11/39) of hospitals by 2015. Evidence from rural health and demographic surveillance sites in Tanzania consistently show that about 30% of deaths occur in health facilities with medical attendance which should then be medically certified. This discrepancy could be because either: i) not all health facilities are reporting in DHIS2, and/or ii) not all deaths in each reporting facility are getting an MCCD recorded in DHIS2. A possible reason for the missing deaths at the hospital level is that several large teaching, speciality (e.g. cancer), and private hospitals were not using DHIS2 because they already had legacy proprietary hospital information systems that were not easily interoperable with DHIS2. In 2018, some large regional referral hospitals were in transition from proprietary electronic medical records systems to a common digital system (Government of Tanzania Hospital Management Information System (GoT-HoMIS) which should be interoperable with DHIS2. The proportion of health facility deaths missing MCCD due to non-reporting facilities is unknown [38]. This is a critical issue as missing data, for example from cancer hospitals, could have important implications for estimating the leading causes of death.

### ICD-10 code ranges

At national level, there was a moderate increase in the number of unique ICD-10 codes used in 2018 (from an average of 109 over the prior 4 years to 324 in 2018). This is possibly an outcome of the national roll-out of MCCD training in 2018. However, there was a much larger increase in unique ICD-10 codes (from 110 to 482) in the Iringa Intervention Region by 2019. This could be an outcome of the more intensive MCCD training across all clinical cadres rather than just two clinicians per facility, combined with the use of the DHIS2 SMoL module introduced in 2019 giving access to a greater range of codes. This is coincident with a decrease in insufficiently specified causes in the Iringa intervention region.

### Quality of cause of death data

#### National baseline

The central measure of quality in the ANACONDA analysis is the prevalence and nature of the unusable and insufficiently specified codes. The proportions of deaths with unusable and insufficiently specified codes seen annually at the national level were remarkably consistent from year to year (five-year means of 24.6 and 16.8% respectively). Fully usable and specified causes were available for policy purposes on only 51.9% of the health facility mortality data at national level. This provides a solid national baseline of quality performance on which to measure the impact of several quality improvement interventions introduced since 2018.

#### After Iringa interventions

Following integrated MCCD interventions at regional level in Iringa, there was a reduction in all four types of unusable causes, the net effect of which was a significant improvement in availability of usable causes from 48 to 65% of coded deaths and a more valid tally of the top 20 causes of death. This increased range of available ICD-10 codes has the potential for allowing greater specificity in identifying the underlying cause of death data. This is supported quantitatively by a one-third reduction in insufficiently specified causes post-intervention. Although we are seeing the effect of a synergy of interventions from the theory of change, it is likely that this particular improvement has been driven by the enhanced training in medical certification. Continued reduction in the use of unusable causes can be targeted by using specific feedback into the in-service training regarding the most frequently misused causes identified by the ANACONDA tool.

The fact that all measures improved substantially within a short period suggests the improvement is consequent to one or more of the various interventions targeted at Iringa. Our theory of change invokes a synergy of eight interventions across four domains. We hesitate to speculate which interventions were the main drivers of change since our study was not designed to unpack this. However, the most prominent interventions that were applied in Iringa were the in-service face-to-face retraining, the task sharing among a broader range of clinicians (in Training Interventions and Process Interventions), the introduction of the DHIS2 SMoL Module (in Practice Interventions), and the MCCD quality reviews using ANACONDA (in Governance Interventions). It is plausible that these contributed most to the observed quality improvement. The observation that there was no improvement in these same measures over 5 years at the national level in the absence of interventions lends plausibility to the intervention effect in Iringa.

### Implications for improving data quality

Overall, these results indicate a continuing need for interventions to improve uCOD data quality in Tanzania through:

1. Increasing the availability of underlying cause of death data;
2. Reducing the prevalence of unusable and insufficiently specified codes;
3. Addressing the under-reporting of injuries and external causes.

### Increasing the availability of data

Our results suggest that approximately 10% of the expected deaths in Tanzania obtain an ICD-10 code of any quality. This represents approximately one-third of the deaths that occur in health facilities with a physician potentially in attendance. Interventions for improving the completeness of mortality statistics would need to ensure that all facilities report all their mortality statistics to the MoHCDGEC via DHIS2 or a compatible/interoperable digital health information system. Any discrepancy between deaths occurring in health facilities with physician attendance, and deaths receiving an MCCD could be addressed by mandating all hospitals to track the total number of deaths at their facilities compared to the number of individual MCCDs reported in DHIS2. Physicians and hospital decision-makers may require additional training on the application of MCCD (i.e. for all facility deaths) and on how to handle challenging cases (e.g. brought in dead).

An analysis of 2002 verbal autopsies for the underlying cause of death in Iringa Region (Nov 2018 to Aug 2019) found that 71% of deaths occurred in the community and only 29% occurred in health facilities (MoHCDGEC unpublished data). The large proportion of deaths in the community will be highly unlikely to have an MCCD certified by a clinician. A large number of deaths outside health facilities where there is no doctor means that the Government misses them in decision-making. Tanzania is now developing a CRVS integrated national verbal autopsy sample system stratified by regions, councils, and wards to complement the selection and other biases seen in health facility-based cause of death data [39].

### Reducing prevalence of unusable and insufficiently specified codes

The problem of unusable codes was skewed to only 15 ICD-10 codes that accounted for almost 78% of the deaths with unusable codes. The top three unusable codes accounted for 48% of these deaths. Hence, pre-service and in-service training or re-training of physicians to avoid using these particular identified codes could rapidly reduce the frequency of unusable codes. This knowledge has been included in targeting the current national MCCD training that commenced in 2019.

The quality of filled certificates has often been questioned and shown to be suboptimal in many countries including high-income countries [36, 40, 41]. In low- and middle-income countries (LMIC) this is of high concern [7] as a large number of health facilities are operated without any physician. Even when a physician is accessible, they may not have the incentive to fill the MCCD form properly, something that is usually considered an administrative task. Even if they have an incentive to fill the MCCD form they might not be adequately trained or experienced to identify the underlying causes of death [36, 42].

One means to improve physician performance in medical certification is the new DHIS2 SMoL module introduced in 2019 that gives on-line access to a large dictionary of possible morbid conditions (including aliases) and distinct ICD-10 codes. As part of the DHIS2 SMoL implementation, there is a training program focused on 26 simplified rules for selecting the uCOD that are applied by physicians or data entry staff to specify the appropriate uCOD [20]. We have shown that this intervention has contributed to a rapid improvement in reducing both unusable and insufficiently specified codes in 2019 and substantially reducing the contribution of high impact unusable causes in the top twenty causes of death.

### Incorporating and improving sources of data on external causes

Globally, external causes of death usually account for 5 to 10% of all deaths, irrespective of whether the country is high-, middle-, or low-income. In a series of deaths in hospitals, we would expect that proportion to be even higher since many victims of traffic crashes and other injuries arrive in hospitals for emergency care. However, across the 5 years of the national data set, the proportion attributed to sufficiently specified external causes never exceeded 1% even though an average of 6.2% (range 5.6 to 6.9%) of ICD-10 codes were for external causes (Chapter XIX - Injury and other consequences of external causes and Chapter XX - External causes). The majority of codes used from these Chapters were unusable garbage codes because many clinicians are coding the trauma at the intermediate cause level rather than identifying the underlying cause. If properly coded, we would know how many are due to specific underlying causes such as road traffic crashes, etc. Clinician training needs to reinforce the need to seek the underlying cause of the trauma and enter it in the MCCD. Furthermore, causes seen in the coronal medico-legal system (e.g. assault, self-harm, road traffic) may remain in those silos and not always added to the MCCD data in the DHIS2 or CRVS system.

The use of ANACONDA in Tanzania supports a new emphasis on improved training and establishing sustainable quality assurance and performance improvement mechanisms is now underway. Efforts are also underway to incorporate data from Tanzania’s teaching and referral hospitals, the medico-legal death investigation system, including data on road traffic fatalities, and other external causes as well as capturing deaths in the community with verbal autopsy. Tanzania is exploring all of these interventions to improve both quantity and quality of cause of death data for policy purposes.

## Conclusions

In 2018, a certified cause of death was available for only 10% of expected deaths at national level in Tanzania and COD medical certification errors were prevalent in almost half of these deaths. This compromises the potential policy value of mortality data at national and sub-national levels and constrains the capacity to monitor the 15 SDG indicators that require cause-specific mortality data.

Synergistic interventions across governance, training, process, and practice domains that combined modern eHealth technologies with targeted training informed by specific quality assessment resulted in important improvements in the quality of medically certified cause of death data detectable within 1 year. Such quality improvements can be cost-effectively and sustainably monitored by periodic (six-monthly) evaluations with the ANACONDA tool at both national and sub-national levels. ANACONDA analyses permit effective evaluation of performance and help the targeting of interventions to remaining weaknesses.

## Supplementary Information

**Additional file 1: Table S1.** Number of expected deaths and proportions with MCCD ICD-10 each year (mainland Tanzania).

**Additional file 2: Figure S1.** Age distribution of deaths according to GBD broad causes and unusable code severity level for the most recent year (2018). Red = Group 1 Communicable, maternal, neonatal and nutritional conditions. Blue = Group 2 Non-communicable diseases. Green = Group 3 External causes including injury. Grey = Unusable causes of varying levels of severity.

**Additional file 3.** Tanzania ANACONDA *.mqr files for 2014–2019.

## Data Availability

ANACONDA analytic files for each year are available online as supplemental information files with this publication.

## Abbreviations

5q0: Probability of death between birth and age 5 years
ANACOD: Analysis of Cause of Death MS Excel software application
ANACONDA: Analysis of Cause of Death for National Action Java software application
COD: Cause of Death
CDR: Crude death rate
CPD: Continuing Professional Development
CRVS: Civil Registration and Vital Statistics
CSMF: Cause-specific mortality fraction
DHIS2: District Health Information System 2
GBD: Global Burden of Disease
GoT-HoMIS: Government of Tanzania Hospital Management Information System
HMIS: Health Management Information System
ICD-10: WHO International Statistical Classification of Diseases and Related Health Problems, 10th Revision
ICD-10-SMoL: WHO ICD-10-Startup Mortality List
MCCD: WHO standard Medical Certificate of Cause of Death
MoHCDGEC: Tanzania Ministry of Health, Community Development, Gender, Elderly and Children
NBD: National Burden of Disease
NBS: National Bureau of Statistics
PPP: Purchasing Power Parity
SDG: UN Sustainable Development Goals
uCOD: Underlying cause of death
