# Supplementary figures and images for: Improving quality of medical certification of causes of death in health facilities in Tanzania 2014–2019
Source: BMC Health Serv Res. 2021 Sep 13;21(Suppl 1):214. doi: 10.1186/s12913-021-06189-7 (PMC8436444; doi:10.1186/s12913-021-06189-7)

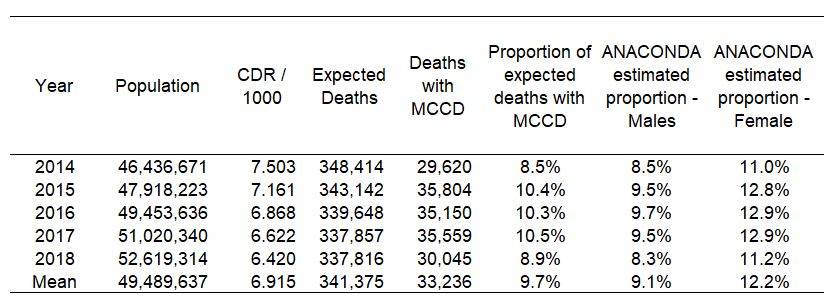

Supplement: Supplementary file 1 — Additional file 1: Table S1. Number of expected deaths and proportions with MCCD ICD-10 each year (mainland Tanzania). [file 12913_2021_6189_MOESM1_ESM.jpg]

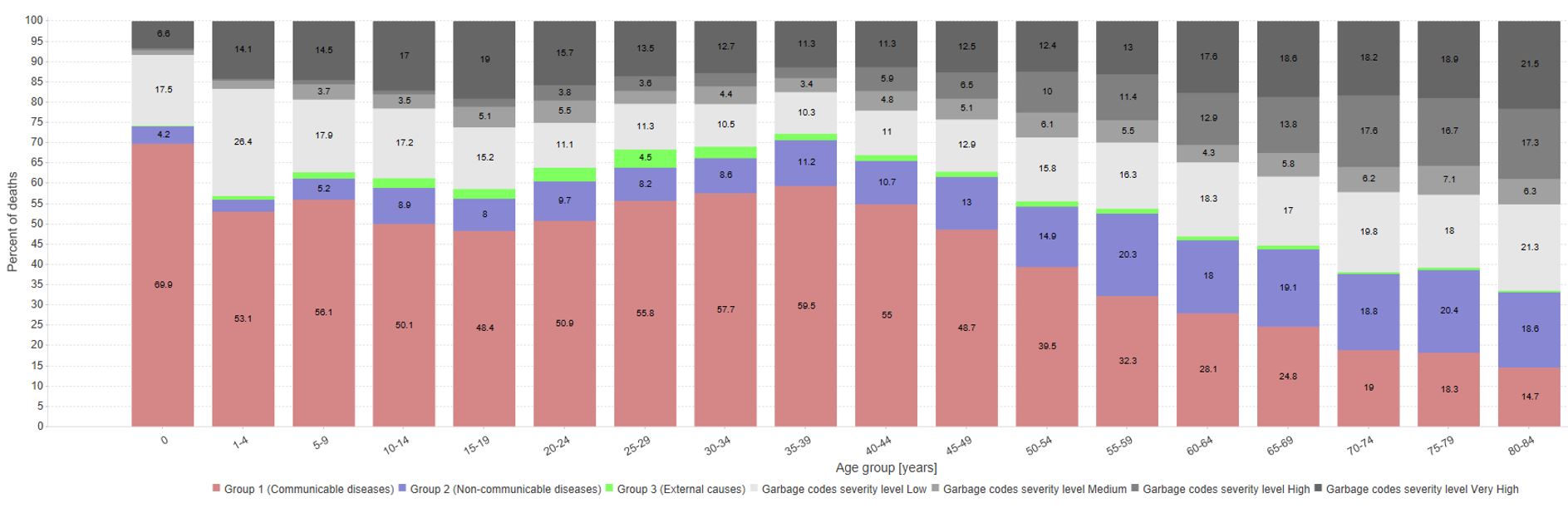

Supplement: Supplementary file 2 — Additional file 2: Figure S1. Age distribution of deaths according to GBD broad causes and unusable code severity level for the most recent year (2018). Red = Group 1 Communicable, maternal, neonatal and nutritional conditions. Blue = Group 2 Non-communicable diseases. Green = Group 3 External causes including injury. Grey = Unusable causes of varying levels of severity. [file 12913_2021_6189_MOESM2_ESM.jpg]
